# Comparison of Cost-Effectiveness Between Digital Health Interventions and Pharmacotherapy for Depression: Systematic Review

**DOI:** 10.2196/70248

**Published:** 2025-09-10

**Authors:** Jiae Im, Byeong-Chan Oh, Ha-Jun Song, Jeong-Min Choi, Dong-Ho Yeo, Eui-Kyung Lee

**Affiliations:** 1 School of Pharmacy Sungkyunkwan University Gyeonggi-do Republic of Korea

**Keywords:** depression, digital health intervention, pharmacotherapy, economic evaluation, systematic review

## Abstract

**Background:**

Owing to the unique characteristics of digital health interventions (DHIs), a tailored approach to economic evaluation is needed—one that is distinct from that used for pharmacotherapy. However, the absence of clear guidelines in this area is a substantial gap in the evaluation framework.

**Objective:**

This study aims to systematically review and compare the economic evaluation literature on DHIs and pharmacotherapy for the treatment of depression.

**Methods:**

We searched for articles published between January 2013 and October 2023 in Ovid MEDLINE, Embase, Cochrane Library, and PsycINFO databases. Studies were eligible if they evaluated DHIs or pharmacotherapies for depression and reported economic outcomes. We extracted data on the study characteristics, input parameters, and economic evaluation modeling components. Chi-square tests were used to analyze the frequency of various components across intervention types. A qualitative comparison was performed to assess the costs, effects, and modeling aspects of each intervention. The Consolidated Health Economic Evaluation Reporting Standards (CHEERS) checklist was used to evaluate the quality of the selected studies.

**Results:**

A total of 42 articles were included, of which 23 (23/42, 55%) focused on DHIs and 19 (19/42, 45%) on pharmacotherapy. Cost-utility analysis was used more frequently in pharmacotherapy (16/19, 84%) than in DHIs (12/23, 52%), with a significant difference between the 2 intervention types (*P*=.01). Similarly, the types of comparators differed significantly, with DHIs more often being compared to usual care (12/23, 52%) or waitlist controls (5/23, 22%) and pharmacotherapy studies mainly involving active controls (17/19, 89%; *P*<.001). In addition, pharmacotherapy was more likely to be used in model-based studies (13/19, 68%), whereas DHIs predominantly relied on trial-based studies (17/23, 74%; *P*=.006). Although not statistically significant (*P*=.28), a notable trend was observed: the payer perspective was most commonly applied in pharmacotherapy studies (10/19, 53%), compared with approximately 30% (7/23) in DHIs. Furthermore, studies with a time horizon exceeding 12 months were more common for pharmacotherapy (5/19, 26%) than for the DHIs (3/23, 13%). Assessment using the CHEERS checklist indicated that pharmacotherapy studies generally had higher reporting quality compared with the quality of DHI studies in areas such as study parameters, comparators, time horizon, and discount rate.

**Conclusions:**

Compared with pharmacotherapy, DHIs involved a higher proportion of trial-based studies reporting short-term outcomes and studies with ambiguously defined cost items. This underscores the need for improved measurement and modeling to accurately capture the costs and effectiveness of DHIs.

**Trial Registration:**

PROSPERO CRD42023471565; https://www.crd.york.ac.kr/PROSPERO/wiew/CRD42023471565

## Introduction

### Background

The rapid advancement of digital technologies in the health care sector has been substantially driven by public health emergencies and other significant health-related events, such as the COVID-19 pandemic. This has led to the emergence and increasing adoption of digital health [1]. Digital health broadly encompasses the use of information and communication technologies across health care, including mobile and wearable devices, telemedicine, and personalized medicine. Digital health interventions (DHIs) refer to digital therapeutics and software medical devices designed to support or deliver treatment [2]. Compared to traditional pharmaceuticals, digital health solutions offer distinct advantages, including shorter development timelines, lower costs, and minimal safety concerns, making them increasingly attractive options in modern health care [3,4]. The number of clinical trials related to DHIs is increasing, with a considerable proportion of these trials focusing on mental health [5,6]. A notable development in this field is the introduction of digital therapeutics into the market, as exemplified by the recent Food and Drug Administration approval of prescription digital therapeutics aimed at treating major depressive disorders [7]. Although DHIs cover a wide array of applications across various clinical areas, this study centered on treatment-oriented interventions for depression, reflecting the increasing attention to digital therapeutics in mental health.

DHIs can complement or replace traditional therapies. Therefore, evaluating their cost-effectiveness compared with the existing treatment options is crucial [8]. In addition, guidelines for the inclusion of digital therapeutics in several countries [9,10] highlight the necessity to assess both the economic impact and cost-effectiveness of these interventions. For economic evaluation results to guide reimbursement decisions, it is imperative that the methodology used is consistent and robust [11,12]. For pharmaceuticals, economic evaluation results have been consistently used in pricing decisions and determining insurance coverage, and their significance and reliability have been well established. Over the years, the extensive research and development of guidelines have contributed to a relatively robust methodological framework. In contrast, further research in digital health is required to develop standardized approaches [13,14].

There are several key differences among traditional pharmaceuticals, medical devices, and DHIs [8]. In particular, DHIs can impact factors beyond health care and often involve interactive engagement with patients (users) through active participation. These unique characteristics require a different methodological approach to economic evaluation, distinct from that applied to pharmaceuticals [15].

Previous studies revealed several limitations in the existing economic evaluations of depression interventions [16]. Systematic reviews addressing the economic evaluations of depression often do not restrict their scope to digital technologies alone, thus limiting the appropriateness of comparisons between DHIs and pharmacotherapy. In addition, model-based systematic reviews are often confined to studies using modeling, which restricts their comprehensiveness [17,18]. Moreover, previous systematic reviews of economic evaluations on DHIs tend not to focus specifically on depression as an indication and lack methodological comparisons with conventional pharmacotherapy, highlighting key distinctions from this study [19-21].

### Objectives

We conducted a systematic review to compare the methodologies used in the economic evaluation of DHIs and conventional pharmacotherapy. This study aimed to provide a current empirical analysis of these evaluations and offer an in-depth examination of the methodological differences between DHIs and traditional pharmacotherapy.

## Methods

### Overview

The study protocol was registered in PROSPERO (CRD42023471565). While the original protocol planned to compare pharmacotherapy, psychotherapy, and their combination, the scope was refined during the review process to focus on DHIs and pharmacotherapies, reflecting the availability and relevance of economic evaluations. All evaluation methods were based on the recommendations of PRISMA (Preferred Reporting Items for Systematic Reviews and Meta-Analyses) [22]. The completed PRISMA checklist is provided in Multimedia Appendix 1.

### Literature Search

The first author (JI) searched the Ovid-Embase, MEDLINE, PsycINFO, and Cochrane Library databases for eligible studies published between January 1, 2013, and October 16, 2023, using terms related to economic evaluations and depression treatments. This time frame was selected to capture recent methodological trends in economic evaluations of DHIs over the past decade. The year 2013 predates the Food and Drug Administration’s first approval of a prescription digital therapeutic in 2017, marking the beginning of formal regulatory recognition of digital interventions in health care. In addition, the surge in digital health adoption during the COVID-19 pandemic further accelerated the integration of such technologies into clinical and economic evaluations [23]. The following search terms were included: “depression”; “depressive symptom”; “pharmacotherapy”; “digital health intervention”; “cost-effectiveness analysis”; and “economic evaluation.” The complete search strategy for each database is provided in Multimedia Appendix 2.

### Eligibility Criteria

Duplicates were initially identified using the Ovid duplicate function, followed by further removal using EndNote (Clarivate) and manual screening. After deduplication, 2 reviewers (JI and HJS) independently screened the titles and abstracts of the remaining articles using predefined eligibility criteria. They also independently reviewed the full texts of potentially relevant studies. Discrepancies at any stage were resolved through discussion and consensus.

Eligible interventions included DHIs and pharmacotherapies for depression. DHIs that solely modified the mode of delivery without therapeutic content (eg, telemedicine) were excluded. Studies were included if they used these interventions for treating depression and reported economic evaluation outcomes. Detailed inclusion and exclusion criteria are provided in Multimedia Appendix 3.

### Data Extraction

Data were extracted using a standardized data extraction template in Microsoft Excel (version 2016). Data on the following characteristics of the included studies were extracted: author, publication year, country of study, modeling methods, target patients, perspective, interventions, time horizon, discount rate, cost and effectiveness indicator, and sensitivity analysis method. For model-based economic evaluations, we also extracted information related to the modeling approach, including the type of economic evaluation model and the health conditions considered. In addition, cost categories reported in the studies were reclassified to enable consistent comparison across evaluations (see Multimedia Appendix 4 for details). Data extraction was initially conducted by the first author (JI), after which an independent reviewer (HJS) validated the extracted data.

### Quality Assessment

The quality of reporting for all selected studies was evaluated using the Consolidated Health Economic Evaluation Reporting Standards (CHEERS) checklist, which was designed to enhance transparency and rigor in health economic reporting [24]. This checklist comprises 24 items across six categories: (1) title and abstract, (2) introduction, (3) methods, (4) results, (5) discussion, and (6) additional information. The first author (JI) assessed all included studies, and an independent reviewer (Yuna Hong) evaluated a randomly selected subset of 23 (55%) of the 42 studies to ensure consistency. Any discrepancies between reviewers were resolved through discussion to reach consensus. Due to the wide heterogeneity of interventions and the methodological focus of this review, a formal certainty of evidence assessment was not performed.

### Statistical Analysis

We conducted comprehensive quantitative and qualitative analyses to evaluate and compare the economic components and modeling approaches of each intervention. Studies were grouped by intervention type (DHIs vs pharmacotherapies), and only those reporting sufficient modeling and cost-effectiveness data were included in the synthesis. Descriptive statistics, including frequencies and percentages for categorical variables, were used to summarize study characteristics. Chi-square tests were used to identify significant differences in economic evaluation aspects, such as modeling methods, evaluation perspectives, and time horizons, with statistical significance set at 2-sided *P*<.05. To assess potential data duplication, sensitivity analyses excluding studies by the same author published in the same year were conducted. In addition, a qualitative analysis was conducted to compare interventions based on modeling elements that could not be quantitatively measured, such as outcome types and characteristics. All analyses were conducted using Stata software (version 18; StataCorp).

## Results

### Study Selection

**Figure 1:**
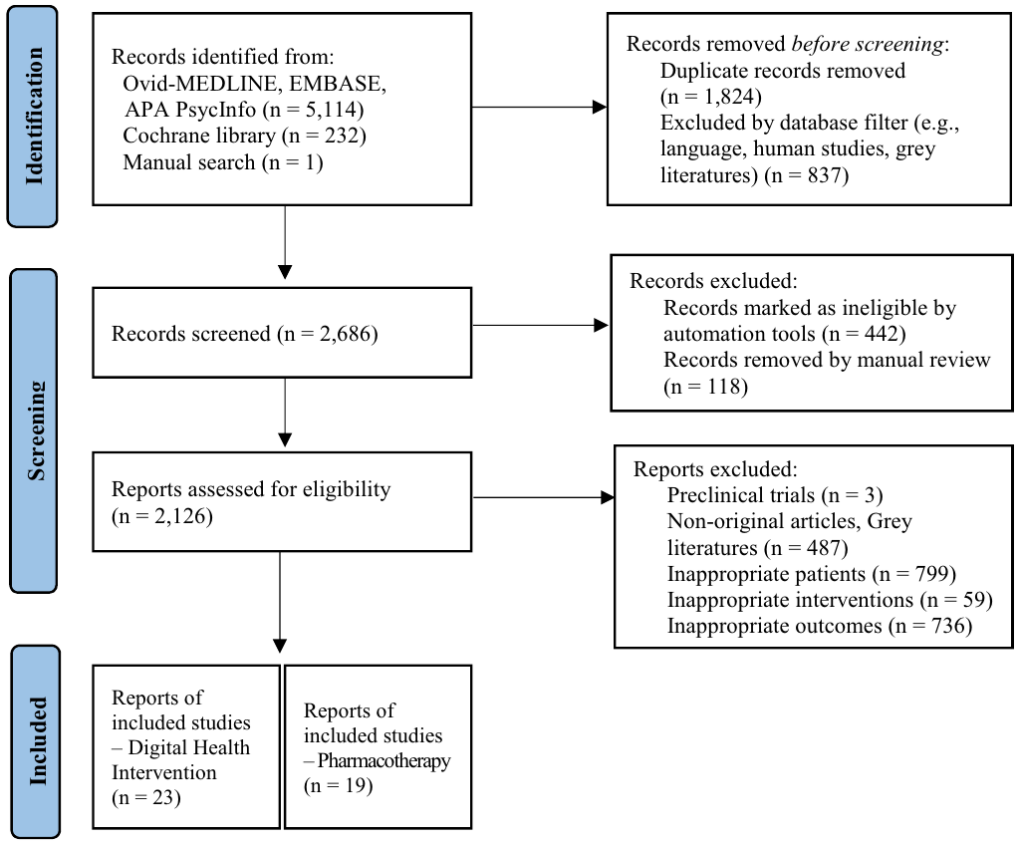
PRISMA (Preferred Reporting Items for Systematic Reviews and Meta-Analyses) flow diagram of the study selection process.

A total of 5347 records were identified through Ovid MEDLINE, Embase, APA PsycINFO (n=5114, 95.64%), the Cochrane Library (n=232, 4.34%), and a manual search (n=1, 0.02%). After removing 2661 (49.77%) duplicate records, 2686 records remained for screening. During the screening process, 560 (20.81%) records were excluded, including 442 (79.1%) marked as duplicates using the EndNote duplicate detection function and deemed ineligible by automation tools, and 118 (21.1%) were removed via manual review. Subsequently, 2126 reports were assessed for eligibility. Reports were excluded for the following reasons: preclinical trials (n=3, 0.14%), nonoriginal articles and gray literature (n=487, 22.91%), noneligible patients (n=799, 37.58%), noneligible interventions (n=59, 2.78%), and noneligible outcomes (n=736, 34.62%). Ultimately, 23 (1.08%) studies focusing on digital interventions and 19 (0.89%) focusing on pharmacotherapy met the inclusion criteria and were included in the final review (Figure 1).

### Study Characteristics and Design

Table 1 summarizes the general characteristics of the included studies, comprising 2 main intervention types: DHIs and pharmacotherapy. A total of 23 studies focused on DHIs, predominantly internet-based cognitive behavioral therapy programs. These studies targeted various patient populations, such as those with mild, moderate, or severe depression, and used diverse comparators, including standard cognitive behavioral therapy, usual care, or waitlist controls. Economic evaluations were conducted from diverse perspectives, including those of health care providers, society, payers, and employers.

**Table 1:** Characteristics of the included studies (N=42).

Intervention type and study		Type	Main perspective	Patients with disease or disorder	Interventions	Comparators	Modeling
**Digital health interventions**							
	Boggs et al [25], 2022	CEA^a^	Health plan	Depression	Mindful Mood Balance and usual care	Usual care	Trial based
	Langergaard et al [26], 2022	CUA^b^	Intervention	MDD^c^	Blended CBT^d^ (online module and face-to-face consultations)	Standard CBT	Trial based
	Piera-Jiménez et al [27], 2021	CUA	Health care provider and societal	Mild, moderate, or severe MDD	Super@ (internet-based CBT)	Usual care	Model based
	Baumann et al [28], 2020	CUA	Societal	Unipolar depression	Internet-based CBT	Face-to-face CBT	Model based
	Gräfe et al [29], 2020	EE^e^	Payer	Mild or moderate depressive disorder	deprexis (online intervention)	Usual care and digital brochure	Trial based
	Richards et al [30], 2020	CUA	Health care and societal	Depression and anxiety disorder	Space from Depression (internet-based CBT)	Waitlist	Trial based
	Thase et al [31], 2020	CUA	Societal	Drug-free MDD	Good Days Ahead program (computer-delivered CBT)	Standard CBT	Trial based
	Kooistra et al [32], 2019	CUA	Health care provider and societal	MDD	Blended CBT (online module and face-to-face consultations)	Standard CBT	Trial based
	Yan et al [33], 2019	CUA	Payer	Depression	Online CBT and TAU^f^	Stepped-care pathway	Model based
	Holst et al [34], 2018	CUA	Health care or payer and societal	Mild to moderate depression	Internet-mediated CBT	Usual care	Trial based
	Kolovos et al [35], 2018	CUA	Societal	Depressive symptoms	Internet-based interventions	Usual care	Trial based
	Kraepelien et al [36], 2018	CUA and CEA	Health care provider or societal	Mild to moderate depression	Internet-based CBT	Usual care	Trial based
	Wijnen et al [37], 2018	CUA and CEA	Health care and societal	Mild to moderate depressive symptoms	Web-based compliant-directed mini interventions	Waitlist	Trial based
	Duarte et al [38], 2017	CUA	Health care	Depression	MoodGYM and Beating the Blues (computerized CBT)	Usual general practitioner care	Trial based
	Lee et al [39], 2017	CUA	Payer	Anxiety and depressive disorders	MindSpot	Usual care	Model based
	Romero-Sanchiz et al [40], 2017	CUA and CEA	Societal	Mild to moderate depression	Smiling is Fun (internet-based CBT)	Usual general practitioner care	Trial based
	Wright et al [41], 2017	CUA	NR^g^	Adolescents with low mood or depression	Stressbusters (computer-administered CBT)	Attention control	Trial based
	Kolovos et al [42], 2016	CUA and CEA	Societal	MDD	Taking Control (internet-based problem-solving self-help treatment)	Enhanced usual care	Trial based
	Geraedts et al [43], 2015	CUA and CEA	Societal and employer	Employees with depressive symptoms	Happy@Work (a worker-directed web-based guided self-help intervention)	Usual care	Trial based
	Solomon et al [44], 2015	CUA	Health care provider	Mild to moderate depression	myCompass (internet-based interventions)	Usual care and face-to-face CBT	Model based
	Titov et al [45], 2015	CUA	National health provider	Depressive symptoms	Therapist-guided internet-delivered CBT	A delayed-treatment waitlist control	Trial based
	Philips et al [46], 2014	CUA	NR	Employees with depressive symptoms	MoodGYM (computerized CBT)	Attention control	Trial based
	Koeser et al [47], 2013	CUA	Health care	Moderate and severe depression	Positive Mental Training and Beating the Blues (computerized CBT)	Usual care	Model based
**Pharmacotherapy**							
	K et al [48], 2023	CEA	Patient	Moderate to severe depression	Escitalopram	Desvenlafaxine	Trial based
	Rognoni et al [49], 2023	CUA	Societal	Treatment-resistant depression	Esketamine	Placebo	Model based
	Atsou et al [50], 2021	CUA	Payer	Patients who did not respond to initial treatment for MDD	Vortioxetine	Levomilnacipran and vilazodone	Model based
	Eldar-Lissai et al [51], 2020	CUA	Payer	Adult patients with postpartum depression	Brexanolone injection	SSRIs^h^	Model based
	Hollingworth et al [52], 2020	CUA	NHS^i^ and personal societal services	Depression or low mood	Sertraline	Placebo	Trial based
	Wang et al [53], 2020	CEA	Chinese	MDD	Vortioxetine	Venlafaxine XR^j^	Trial based
	Rubio-Valera et al [54], 2019	CUA	Health care and government	Mild to moderate MDD	Antidepressants	Active monitoring	Trial based
	Yoon et al [55], 2018	CUA	Health care	Antidepressant-resistant MDD	Switch to bupropion	Augment with bupropion and augment with aripiprazole	Trial based
	Singh et al [56], 2017	CEA	Payer	Patients who did not respond to initial treatment for MDD	Bupropion	Sertraline and venlafaxine	Trial based
	Soini et al [57], 2017	CUA	Payer	MDD with inadequate response to SSRI or SNRI^k^	Vortioxetine	Agomelatine, bupropion SR^l^, sertraline, and venlafaxine XR	Model based
	Young et al [58], 2017	CUA	Payer	MDD with inadequate response to alternative antidepressants	Vortioxetine	Duloxetine, venlafaxine, agomelatine, escitalopram, citalopram, and sertraline	Model based
	Choi et al [59], 2016	CUA	Limited societal	MDD with inadequate response to alternative antidepressants	Vortioxetine	Venlafaxine XR	Model based
	Khoo et al [60], 2015	CUA	Societal	Moderate to severe MDD	Mirtazapine	Fluoxetine, fluvoxamine, paroxetine, sertraline, venlafaxine, escitalopram, Trazodone, agomelatine, and duloxetine	Model based
	Annemans et al [61], 2014	CUA	Payer and societal	MDD undertaking first-line treatment	Venlafaxine	Escitalopram, mirtazapine, sertraline, paroxetine, fluoxetine, citalopram, and duloxetine	Model based
	Maniadakis et al [62], 2013	CUA	Societal	MDD	Agomelatine	Venlafaxine, sertraline, escitalopram, fluoxetine, generic venlafaxine, generic sertraline, generic escitalopram, and generic fluoxetine	Model based
	Mencacci et al [63], 2013	CUA	Payer	MDD	Escitalopram	Sertraline, venlafaxine XR, paroxetine, citalopram, fluoxetine, duloxetine, and fluvoxamine	Model based
	Mencacci et al [64], 2013	CUA	Payer	MDD	Escitalopram	Venlafaxine XR, sertraline, paroxetine, citalopram, fluoxetine, duloxetine, and fluvoxamine	Model based
	Mencacci et al [65], 2013	CUA	Payer	MDD	Escitalopram	Citalopram, paroxetine, and sertraline	Model based
	Saylan et al [66], 2013	CUA	Payer	MDD responding insufficiently to antidepressants	Augment with aripiprazole	Augment with quetiapine and augment with olanzapine	Model based

^a^CEA: cost-effectiveness analysis.

^b^CUA: cost-utility analysis.

^c^MDD: major depressive disorder.

^d^CBT: cognitive behavioral therapy.

^e^EE: economic evaluation.

^f^TAU: treatment as usual.

^g^NR: not reported.

^h^SSRI: selective serotonin reuptake inhibitor.

^i^NHS: National Health Service.

^j^XR: extended release.

^k^SNRI: serotonin and norepinephrine reuptake inhibitor.

^l^SR: sustained release.

Pharmacotherapy-focused studies (n=19) assessed treatments for moderate to severe depression or patients with inadequate responses to initial treatments. These studies evaluated a range of antidepressants, including newer options, such as vortioxetine, brexanolone, and esketamine. Comparators varied widely, including placebos, alternative antidepressants, and augmentation strategies, such as aripiprazole. The perspectives primarily included societal and payer viewpoints, emphasizing both health care and broader economic implications. Additional characteristics of the included studies are provided in Multimedia Appendix 5 [25-66].

### Comparison of Study Characteristics of 2 Interventions

Overall, 42 studies were included in the analysis, comprising 23 (55%) DHI and 19 (45%) pharmacotherapy studies. Pharmacotherapy studies were significantly more likely to involve cost-utility analysis (CUA) as the primary evaluation method (16/19, 84%) compared with DHI studies (12/23, 52%; *P*=.01). Notably, cost-effectiveness analysis (CEA) and CUA were more frequently combined in DHI studies (7/23, 30%), a method not observed in pharmacotherapy studies.

Differences in the comparator choice were also apparent. DHIs were most often compared with usual care (12/23, 52%) or waitlist controls (5/23, 22%), whereas pharmacotherapy studies predominantly used active comparators (17/19, 89%; *P<.*001). The methodological approaches further revealed a contrast: pharmacotherapy studies were more likely to involve model-based evaluations (13/19, 68%), whereas DHI studies predominantly relied on trial-based evaluations (17/23, 74%; *P*=.006).

The payer perspective was predominantly used in pharmacotherapy studies for economic evaluation (10/19, 53%) compared with DHI studies (7/23, 30%). Although this difference was not statistically significant (*P*=.28), it suggests distinct considerations for the evaluation approaches. In addition, pharmacotherapy studies more frequently involved time horizons exceeding 12 months (5/19, 26%) compared with DHI studies (3/23, 13%). A summary of these findings is provided in Table 2.

**Table 2:** Comparison of study characteristics between digital health interventions (DHIs) and pharmacotherapy.

Item and characteristics		DHIs (n=23), n (%)	Pharmacotherapy (n=19), n (%)	*P* value	
**Perspective^a^**					.32
	Payer	7 (30)	10 (53)		
	Health care system	2 (9)	1 (5)		
	Societal	5 (22)	5 (26)		
	Else^b^	9 (39)	3 (16)		
**Type**					.01
	CEA^c^	1 (4)	3 (16)		
	CUA^d^	12 (52)	16 (84)		
	CEA and CUA	7 (30)	0 (0)		
	NR^e^	3 (13)	0 (0)		
**Modeling**					.006
	Model based	6 (26)	13 (68)		
	Trial based	17 (74)	6 (32)		
**Time horizon**					.28
	≤12 mo	20 (87)	14 (74)		
	>12 mo	3 (13)	5 (26)		
**Comparator**					<.001
	Standard	5 (22)	17 (89)		
	Placebo	12 (52)	2 (11)		
	Waitlist	5 (22)	0 (0)		
	Else^f^	1 (4)	0 (0)		
**Funding**					.002
	Company	3 (13)	12 (63)		
	Government	12 (52)	4 (21)		
	University or research funding	6 (26)	0 (0)		
	Else^f^	2 (9)	3 (16)		
**Country**					.55
	Reference^g^	10 (43)	10 (53)		
	Else	13 (57)	9 (47)		

^a^Classified analysis perspective using a broad concept.

^b^>1 perspective or not reported.

^c^CEA: cost-effectiveness analysis.

^d^CUA: cost-utility analysis.

^e^NR: not reported.

^f^≥2 comparisons.

^g^A8 countries (the United States, the United Kingdom, Germany, France, Italy, Switzerland, Japan, and Canada), which are used as reference countries in Korea for foreign drug pricing.

### Key Differences Between 2 Interventions in the Economic Evaluation of Depression

Through a systematic review of the literature, key differences between the economic evaluation approaches used for DHIs and pharmacotherapies in the treatment of depression were identified. DHIs predominantly relied on trial-based evaluations [25,26,29-32,34-38,40-43, 45-46], which focused on intermediate outcomes and shorter time horizons. These studies frequently used simpler modeling techniques, such as decision trees [33,39,44,47], and often adopted broader perspectives [27,28,30-32,34-37,40,42,43] (many studies incorporating multiple perspectives, at least one of which included societal costs, such as productivity losses, informal caregiving, or broader economic impacts). In contrast, pharmacotherapy was primarily evaluated using model-based approaches [49-51,57-66], emphasizing the final outcomes and extended time frames. These evaluations commonly used Markov models [49-51,57-59,62], incorporating diverse health states and transition probabilities, and were predominantly conducted from a payer perspective [50,51,56-58,61,63-66].

The cost considerations differed between the 2 interventions. DHIs often exhibited variability in cost definitions and classifications, with intervention costs being frequently undefined or excluded [25,29,33,45,46]. In addition, the categorization of these costs often remained unclear, particularly for expenses, such as overhead costs (eg, licensing fees and technical support costs), which were sometimes classified as direct medical costs, but were more commonly considered nonmedical costs [25,26,28-31,34-39,41,43,44,47]. In contrast, pharmacotherapies primarily involved fixed intervention costs with additional expenses for adverse event (AE) management [50,57-59,62] and severe outcomes, such as hospitalization due to suicide attempts, as needed [57,59,61,63-66].

In addition, effectiveness measures reflected different aspects of focus. DHI studies primarily involved intermediate outcome assessments, such as changes in symptom scores (eg, Patient Health Questionnaire-9 and Center for Epidemiologic Studies Depression Scale) [29,30,32-46], while final outcomes (eg, remission rates) were the focus of pharmacotherapy studies [48-51,53,55-66]. These differences in focus influenced the estimation methods used. Quality weights were exclusively established in pharmacotherapy studies using preference-based instruments, such as EQ-5D, considering the disutility associated with AEs and severe outcomes [49-52,54,55,57-66]. In DHI studies, quality weights were sometimes derived using preference-based tools but more commonly estimated utility values drawn from previous literature or symptom-based metrics [27,31,37] (Table 3).

**Table 3:** Key differences between digital health interventions (DHIs) and pharmacotherapies in the economic evaluation of depression.

Items	DHI	Pharmacotherapy
Modeling	Were mostly trial-based studies Used a simplistic structure, such as a decision tree model, with a short analysis period Presented multiple analytical perspectives, including a societal perspective	Were mostly model-based studies Used a complex structure, such as a Markov model, with a relatively long analysis period, various health states, and transition probabilities, including scenario sensitivity analysis Commonly applied the payer perspective
Cost	Often exhibited variability in the definition and classification of costs Intervention costs were yet to be determined or are not included Cost categorization was unclear and varied depending on the articles, for example, direct medical costs sometimes include all expenses related to performing DHIs, including overhead costs, which are classified as nonmedical costs Costs did not consider AEsa	Typically involved a fixed unit price Intervention costs were fixed; additional costs for pharmacotherapy were not incurred Cost categorization was clear Often included costs for managing AEs or hospitalization due to suicide attempts
Comparator	Were commonly used as an add-on therapy to usual care (eg, psychotropic medication and outpatient mental health services).	Were mostly compared to an active control (eg, frequently used medication)
Effectiveness	Generally involved intermediate outcomes, such as improvements in clinical indices (eg, Patient Health Questionnaire-9, Beck Depression Inventory-II, and Center for Epidemiologic Studies Depression Scale)	Generally used final outcomes, such as remission rates
Utility	Often estimated changes in quality of life by calculating effect sizes for symptom scores; rarely considered disutility	Typically used patient-assigned health state utility or preference-based health-related quality of life instruments, considering disutility

^a^AE: adverse event.

### Reporting Quality Assessment

**Figure 2:**
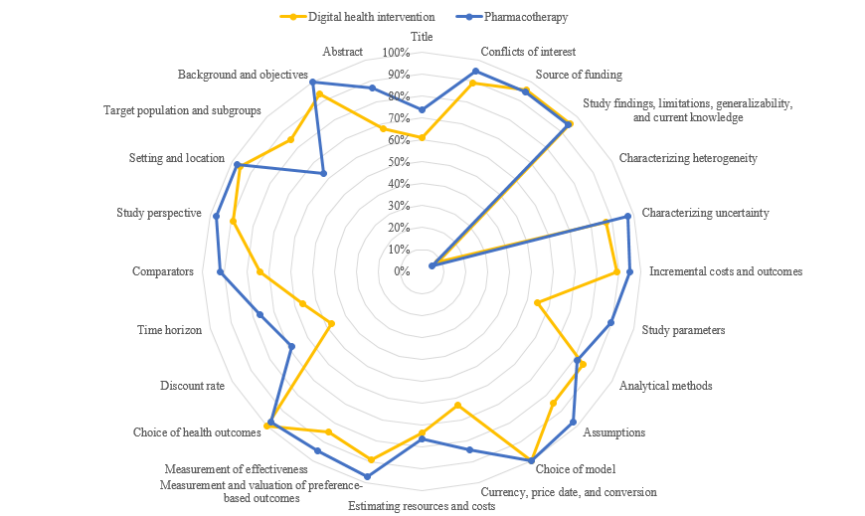
Consolidated Health Economic Evaluation Reporting Standard (CHEERS) checklist results.

The CHEERS checklist evaluation (Figure 2) indicated that pharmacotherapy studies achieved higher scores than DHI studies across key domains, including study design, comparators, time horizon, and discounting. The DHI studies scored lower on parameters related to transparency and methodological rigor, whereas pharmacotherapy studies showed more consistent and higher scores across the assessed categories. These differences highlighted a quantitative disparity in the reporting quality between the 2 intervention types.

## Discussion

This study systematically reviewed and compared the economic evaluations of DHIs and pharmacotherapies for depression, a mental health area in which digital interventions are actively being developed.

### Principal Findings

The analyses revealed distinct differences in the economic evaluation of DHIs and pharmacotherapies. No previous studies have directly compared the component frequencies of DHIs and pharmacotherapies, making direct comparisons with our findings challenging. However, a recent systematic review of economic evaluations for depression by Belay et al [16] reported that CUA was the most frequently used method, and a systematic review on internet and mobile interventions for mental health by Kählke et al [20] found that most selected studies used CUA or CEA. The significant difference in economic evaluation types—particularly the more frequent use of CUA in pharmacotherapies compared to DHIs—may be partly explained by the limited application of utility outcome measures in DHI studies. While pharmacotherapy studies often used preference-based instruments (eg, EQ-5D) to directly estimate utility weights, DHI studies tended to rely on previously published literature or symptom-based indicators instead. This reliance on secondary or non–preference-based data reflects the relatively lower methodological maturity of economic evaluations in DHIs, which may have constrained the application of rigorous CUA in this field. Another significant finding was the use of modeling, where model-based evaluations were predominantly used in pharmacotherapies, but trial-based evaluations were more common in DHIs. This result can be attributed to the inherent characteristics of DHIs, where long-term clinical outcomes are often lacking, and essential components, such as cost, are subject to considerable uncertainty compared with those in pharmacotherapies [67]. This may be partly due to the earlier methodological maturity and established expectations for modeling long-term outcomes in pharmacotherapy, whereas DHI evaluations may still be evolving in this regard.

Qualitative analysis further highlighted this distinction, as it allowed the detailed comparison of intervention characteristics that were difficult to quantitatively assess. Pharmacotherapy often incorporates complex structures with long-term time horizons and diverse health states, whereas DHIs tend to use simpler, shorter-term models. Unlike pharmacotherapy, where costs, such as medication or hospitalization fees, are well defined and typically calculated as fixed costs, DHIs involve unique cost components, including licensing fees, maintenance expenses, and health care provider training. This requires precise estimation to reduce uncertainty and accurately capture the economic impacts of DHIs. Given these differences, further research should explore model-based evaluations that not only leverage diverse modeling scenarios for pharmacotherapies but also account for the distinct characteristics of DHIs, such as their cost structures, rapid technological advancements, and user engagement dynamics.

From the perspective of effectiveness, DHIs commonly rely on intermediate outcomes (eg, improvements in clinical indices), whereas pharmacotherapy often focuses on the final outcomes (eg, remission). The reliance on short-term outcomes can lead to incomplete or potentially distorted evaluations. In addition, the lack of clarity in cost analysis for DHIs can undermine the reliability of CEA, hindering evidence-based decision-making. As uncertainty is inherently higher in DHIs than in pharmacotherapy, a broader integration of real-world evidence (RWE) data from diverse sources could be a viable solution. The application of RWE can be particularly useful in both initial health technology assessment and reassessment processes [68]. Incorporating real-world data, methods for capturing patient-reported outcomes, and frameworks that evaluate multiple dimensions of value may provide a more comprehensive and reliable foundation for evaluating the DHIs.

Moreover, unlike pharmacotherapy, which often includes disutility factors related to adverse effects, limited information exists on the side effects of DHIs and their impact on utility, such as potential sensory or cognitive strain caused by digital tools [69]. Further research on these effects and their implications is required. Finally, DHIs may also have nonhealth effects, such as improved accessibility, leading to increased social participation [8]. Identifying and measuring these dimensions is crucial for developing a comprehensive economic evaluation of DHIs.

The reporting quality of the selected studies for each intervention was assessed using the CHEERS tool, and the scores were compared accordingly. Overall, pharmacological interventions demonstrated higher reporting quality than DHIs, particularly in areas such as study parameters, comparators, time horizons, and discount rates. This may be attributed to the predominance of short-term trial-based economic evaluations in digital health studies. To enhance the reporting quality in economic evaluation studies of DHIs, it is important to consider strategies that emphasize transparency and consistency. These may include providing detailed documentation of the intervention design, implementation processes, and evaluation contexts as well as ensuring clear reporting of cost and outcome measurement methods specific to digital health. These efforts could support more robust and reliable assessments of the economic impacts of such interventions.

### Comparison With Prior Work

Currently, there are no standardized methodological guidelines for the economic evaluation of DHIs. However, Gomes et al [8] provided recommendations for such evaluations, emphasizing that traditional economic evaluation approaches may not be suitable because of the unique characteristics of DHIs. Economic evaluations of digital health should consider intervention-specific characteristics, account for nonhealth impacts, adopt broader analytic perspectives, and conduct cost-consequence analyses [8]. In a comprehensive review of analytic frameworks and outcome measures for DHIs, Benedetto et al [70] recommended assessing equity impacts in these evaluations. Upon examining the alignment of the digital health economic evaluations in this study with recommendations from previous studies, we found limited adherence. Specifically, the DHIs in our sample did not demonstrate a notably higher adoption of broader perspectives (such as societal perspectives) compared with those in pharmacotherapy nor did they commonly account for nonhealth impacts or conduct cost-consequence analyses. However, as these findings are based on the evaluation of specific indications, further research across diverse contexts is warranted to verify these results.

Unlike previous studies, this study conducted a comparative analysis of the actual economic evaluation methods used in pharmacotherapy and DHIs, including CEA, analytic perspectives, and modeling techniques. The findings indicate that compared with pharmacotherapy, DHIs often rely on short-term clinical trial results (intermediate outcomes) and exhibit variability in cost components, leading to unclear cost estimates. Therefore, further research on modeling approaches tailored to DHIs and the development of evidence for long-term outcome measures using real-world data is required. Given the unique characteristics of DHIs, such as their potential nonhealth impacts, it is essential to establish measures that can effectively capture and reflect these dimensions. Finally, when evaluating the reporting quality of economic evaluations, it is crucial to propose frameworks that address the limitations inherent in DHIs, including constraints on modeling and reporting clarity. These efforts will help advance the reliability and comprehensiveness of the economic evaluations of DHIs.

While this study focused on therapeutic DHIs for depression, the methodological limitations identified in the economic evaluation of DHIs are not unique to this area. Similar issues have been reported in previous studies on another major category of digital health—clinical decision support systems (CDSSs). There was notably insufficient reporting of cost components, limited use of model-based analyses, and difficulty isolating intervention effects. In particular, both CDSSs and DHIs exhibit considerable inconsistency in the definition, classification, and inclusion of costs. For CDSSs, implementation-related expenses, such as electronic health record integration, training, and maintenance, are often poorly defined or omitted. Similarly, DHI studies frequently exclude intervention costs altogether or include them without clear categorization; for example, overhead or platform fees may be inconsistently treated as either direct medical or nonmedical costs. Moreover, costs associated with AEs, which can significantly influence utility estimates, are seldom incorporated. These inconsistencies make it difficult to generate comparable findings and may undermine confidence in cost-effectiveness estimates. While pharmacotherapy evaluations typically apply standardized cost definitions and use preference-based instruments such as EQ-5D, digital health studies often rely on short-term outcomes and secondary data sources. To strengthen the validity of future economic evaluations in digital health, consistent cost definitions, explicit inclusion of all relevant cost categories (including AEs), and greater use of modeling and RWE are needed. In addition, improving transparency in cost reporting and adopting standardized evaluation frameworks were common recommendations across previous studies [71,72]. Our study contributes to this evolving field by directly comparing DHIs and pharmacotherapy in depression, offering practical insights into current methodological gaps.

### Limitations

This study had several limitations. First, although our review included 42 studies specifically addressing depression, the applicability of the findings to other mental health disorders remains limited. Second, only English-language publications were considered, which potentially excluded studies published in other languages. Third, the types of DHIs examined were limited, as various intervention forms, such as applications and virtual reality, were not included because of a lack of relevant studies. Finally, by using a systematic review methodology, this study was limited to the published literature, suggesting the need for further exploration of a broader range of diseases and intervention types in future research.

### Conclusions

Compared with pharmacotherapy, DHIs showed a higher reliance on trial-based studies with short-term outcomes and greater variability in cost components, often lacking clear definitions. These findings highlight the need for refined measurement tools and modeling approaches that account for the unique characteristics of DHIs, including their potential long-term impacts and nonhealth benefits. Future research should prioritize the development of comprehensive evaluation frameworks, standardized cost definitions, and the integration of outcomes from diverse sources such as RWE, patient-reported outcomes, and administrative data. Such approaches will enable more robust and comprehensive economic evaluations, ensuring the relevance of DHIs in evidence-based decision-making.

## Appendix

Multimedia Appendix 1 PRISMA checklist.

Multimedia Appendix 2 Database search strategies.

Multimedia Appendix 3 Summary of inclusion and exclusion criteria.

Multimedia Appendix 4 Reclassification of cost categories in the economic evaluations in this review.

Multimedia Appendix 5 Additional characteristics of included studies.

## Abbreviations

AE: adverse event
CDSS: clinical decision support system
CEA: cost-effectiveness analysis
CHEERS: Consolidated Health Economic Evaluation Reporting Standards
CUA: cost-utility analysis
DHI: digital health intervention
PRISMA: Preferred Reporting Items for Systematic Reviews and Meta-Analyses
RWE: real-world evidence

## Data Availability

All data generated or analyzed during this study are included in this published article and its multimedia appendices.
