# Supplementary material for: Comparison of Cost-Effectiveness Between Digital Health Interventions and Pharmacotherapy for Depression: Systematic Review
Source: J Med Internet Res. 2025 Sep 10;27:e70248. doi: 10.2196/70248 (PMC12461167; doi:10.2196/70248)
Supplement: Multimedia Appendix 2 [file jmir_v27i1e70248_app2.pdf]

Multimedia Appendix 2. Database search strategies

No.	Terms
1	exp *Depression/ or depression.mp.
2	(depressive adj2 (mood* or symptom* or disorder*)).mp.
3	*Depressive Disorder, Major/ or major depression disorder.mp.
4	MDD.mp.
5	1-4/OR
6	((pharmaceu* or pharmaco*) adj2 (treatment* or therap*)).mp.
7	SSRI.mp. or exp *Selective Serotonin Reuptake Inhibitors/
8	(escitalopram or fluoxetine or paroxetine or sertraline).mp.
9	SNRI.mp. or exp *Serotonin and Noradrenaline Reuptake Inhibitors/
10	(desvenlafaxine or duloxetine or milnacipran or venlafaxine).mp.
11	(Mirtazapine or Agomelatine or Bupropion or Vortioxetine or Clomipramine or Imipramine).mp.
12	(Tri-or tetra-cyclic antidepressants or TCA).mp.
13	6-12/OR
14	exp *Psychotherapy/ or Psychotherapy.mp.
15	exp *Cognitive Behavioral Therapy/ or CBT.mp.
16	((Behavior* or cognitive or mindfulness or interpersonal or psychodynamic or supportive) adj2 (therap* or treatment* or intervention*)).mp.
17	14-16/OR
18	((digital* or web* or computer* or app* or electronic* or cyber* or text* or tele* or mobile* or phone* or online* or internet*) adj2 (based or deliver* or media* or platform* or technolog* or intervention* or treatment* or thearp*)).mp.
19	(DHI* or DMHI*).mp.
20	18-19/OR
21	13 OR 17 OR 20
22	(cost* adj2 (analys* or impact* or benefit* or utilit* or effective* or estiamte* or saving* or model*)).mp.
23	(economic* adj2 (evaluation* or analys* or impact* or effect* or model* or value*)).mp.
24	22 OR 23
25	5 AND 21 AND 24
26	limit 25 to yr="2013 -Current"
27	remove duplicates from 26
28	limit 27 to english language
29	limit 28 to human
30	limit 29 to (conference abstract or conference paper or "conference review" or editorial or erratum or letter or note or published erratum or technical report) [Limit not valid in Embase,Ovid MEDLINE(R),APA PsycInfo; records were retained]
31	29 NOT 30
