# Supplementary material for: Comparison of Cost-Effectiveness Between Digital Health Interventions and Pharmacotherapy for Depression: Systematic Review
Source: J Med Internet Res. 2025 Sep 10;27:e70248. doi: 10.2196/70248 (PMC12461167; doi:10.2196/70248)
Supplement: Multimedia Appendix 3 [file jmir_v27i1e70248_app3.pdf]

Multimedia Appendix 3. Summary of inclusion and exclusion criteria

Study characteristic	Inclusion criteria	Exclusion criteria
Population	Patients with a depressive disorder or current substantial depressive symptoms	Depressive symptoms secondary to another primary condition (e.g., substance abuse, schizophrenia)
Interventions	Digital health interventions and pharmacotherapy for treatment	Non-digitalized interventions
Comparators	Active comparators or control (e.g., usual care or placebo)	None
Outcomes	All outcomes related to cost-effectiveness analysis of interventions	Only effectiveness outcomes
Time	Studies published from 2013 to 2023	None
Study design	Economic evaluations; cost-effectiveness analyses, cost-utility analyses, and cost-benefit analyses	None
Limits	English language only, original article only	None
