# Supplementary material for: Comparison of Cost-Effectiveness Between Digital Health Interventions and Pharmacotherapy for Depression: Systematic Review
Source: J Med Internet Res. 2025 Sep 10;27:e70248. doi: 10.2196/70248 (PMC12461167; doi:10.2196/70248)
Supplement: Multimedia Appendix 4 [file jmir_v27i1e70248_app4.pdf]

Multimedia Appendix 4. Reclassification of cost categories in the economic evaluations in this review

Items	Definition	Examples
Direct medical costs	Formal and informal medical costs related directly with a health care intervention	Medication, inpatient/outpatient costs, laboratory, physician visits, program-related costs (e.g. monthly license fee, and software maintenance costs)
Direct non-medical costs	Additional costs in accessing a health care intervention but not related to medical treatment or services	Transportation, accommodation, caregiving costs, overhead costs (computer, internet access fee, rental of office space, and cleaning), training costs
Productivity costs	Costs associated with the loss of productivity or opportunities due to illness or treatment	Absenteeism and presenteeism

Adapted from Sittimart et al [1], 2024; Kim et al [2], 2022

1. Sittimart M, Rattanavipapong W, Mirelman AJ, Hung TM, Dabak S, Downey LE, et al. An overview of the perspectives used in health economic evaluations. Cost Eff Resour Alloc. 2024;22:41. doi:10.1186/s12962-024-00552-1
2. Kim Y, Kim Y, Lee HJ, Lee S, Park SY, Oh SH, et al. The Primary Process and Key Concepts of Economic Evaluation in Healthcare. J Prev Med Public Health. 2022;55(5):415-423. PMID: 36229903
