# Supplementary material for: Comparison of Cost-Effectiveness Between Digital Health Interventions and Pharmacotherapy for Depression: Systematic Review
Source: J Med Internet Res. 2025 Sep 10;27:e70248. doi: 10.2196/70248 (PMC12461167; doi:10.2196/70248)
Supplement: Multimedia Appendix 5 [file jmir_v27i1e70248_app5.pdf]

Multimedia Appendix 5. Additional characteristics of included studies

| Author (year) | Country | Modeling | Time horizon (cycle length) | Outcome measure | Sensitivity, Scenario analysis | ICER[a] | WTP[b] threshold |
|---|---|---|---|---|---|---|---|
| *Digital Health Interventions* | | | | | | | |
| Boggs et al [1], 2022 | U.S.[c] | Trial-based | 12-month (None) | Depression free days | Scenario | \$9.63 to \$15.04/DFD[d] for different inputs | at different WTP threshold |
| Langergaard et al [2], 2022 | Denmark | Trial-based | 6-month (None) | QALYs[e] | PSA[f], Scenario | £7,767.50/QALY | £20,000 to £30,000 |
| Piera-Jimenez et al [3], 2021 | Spain | Model-based (Markov) | Lifetime (1-year) | Remission and depression rate, QALYs | DSA[g], PSA, Scenario | Healthcare: €29,367/QALY Societal: €26,484/QALY | €30,000 |
| Baumann et al [4], 2020 | Germany | Model-based (Cohort-based/ Markov) | 3-year (1-week) | Proportion of remission or discontinuing patients, QALYs | DSA, PSA, Scenario | Dominant | €8,580 to €18,420 |
| Gräfe et al [5], 2020 | Germany | Trial-based | 9-month (None) | Depression severity, self-reported functional impairment, EQ-5D-3L[i], SF-12[j] | N.R.[h] | N.R. | N.R. |
| Richards et al [6], 2020 | U.K.[k] | Trial-based | 12-month (None) | Depression severity, Proportion of significant improvement and recovery, Self-reported functional impairment, EQ-5D-5L | Scenario | ITT[l]: £29,764/QALY CC[m]: £25,123/QALY | £20,000 to £30,000 |
| Thase et al [7], 2020 | U.S. | Trial-based | 6-month (None) | Depression severity, Remission rate, QALYs | PSA | Dominant(cost saving: \$928 per patient) | \$50,000 |

| Author (year) | Country | Modeling | Time horizon (cycle length) | Outcome measure | Sensitivity, Scenario analysis | ICER[a] | WTP[b] threshold |
|---|---|---|---|---|---|---|---|
| Kooistra et al [8], 2019 | Netherlands | Trial-based | 30-week (None) | Depression severity, QALYs | Scenario | Healthcare: €-29,333/QALY Societal: €185,880/QALY | at different WTP threshold |
| Yan et al [9], 2019 | Canada | Model-based (Decision-tree) | 1-year (None) | Depression severity, QALYs | PSA, Scenario | $57,113/QALY | $50,000 |
| Holst et al [10], 2018 | Sweden | Trial-based | 12-month (None) | Depression severity, QALYs | DSA, Scenario | Healthcare: €1,109/QALY Societal: €7,395/QALY | €30,000 |
| Kolovos et al [11], 2018 | Netherlands | Trial-based | 12-month (None) | Depression severity, response rates, QALYs | Scenario | 8-week: €81,155/QALY 6-month: €32,706/QALY 12-month: €-1,122,646/QALY | €24,000 to €35,000 |
| Kraepelien et al [12], 2018 | Sweden | Trial-based | 12-month (None) | Depression severity, QALYs | PSA | Primary 3-month, Healthcare provider: €8,817/QALY During 1-year follow-up, Societal: €31,417/QALY | £20,000/ healthcare perspective |
| Wijnen et al [13], 2018 | Netherlands | Trial-based | 6-month (None) | Proportion of responder, QALYs | Scenario | Dominant(€-2,037/QALY) | €20,000 |
| Duarte et al [14], 2017 | U.K. | Trial-based | 24-month (None) | Depression severity, QALYs | PSA, Scenario | Beating the Blues: Dominant(£-3,715/QALY) MoodGYM: £6,933/QALY | £20,000 |

| Author (year) | Country | Modeling | Time horizon (cycle length) | Outcome measure | Sensitivity, Scenario analysis | ICER[a] | WTP[b] threshold |
|---|---|---|---|---|---|---|---|
| Lee et al [15], 2017 | Australia | Model-based (Decision-tree) | 1-year (None) | Depression severity, QALYs | DSA, Scenario | Excluding hospitalization costs: dominant ($-849/QALY) Including hospitalization costs: dominant ($-20,934/QALY) | $50,000 |
| Romero-Sanchiz et al [16], 2017 | Spain | Trial-based | 12-month (None) | Depression severity, QALYs | Scenario | ITT: totally self-guided €-5,160/QALY, low-intensity therapist-guided €497/QALY CC: totally self-guided €-11,390/QALY, low-intensity therapist-guided €-6,381/QALY | €21,000 to €25,000 |
| Wright et al [17], 2017 | U.K. | Trial-based | 4-month (None) | Depression severity, QALYs | N.R. | N.R. | £20,000 to £30,000 |
| Kolovos et al [18], 2016 | Netherlands | Trial-based | 12-month (None) | Depression severity, response rates, QALYs | Scenario | Societal: €157,900/QALY Mental healthcare provider: €-21,800/QALY National healthcare provider: €-3,800/QALY | €20,000 to €30,000 |
| Geraedts et al [19], 2015 | Netherlands | Trial-based | 12-month (None) | Depression severity, QALYs | Scenario | Societal: €314/QALY Employer: €24/QALY | €20,000 to €30,000 |

| Author (year) | Country | Modeling | Time horizon (cycle length) | Outcome measure | Sensitivity, Scenario analysis | ICER[a] | WTP[b] threshold |
|---|---|---|---|---|---|---|---|
| Solomon et al [20], 2015 | Australia | Model-based (Decision-analytic) | 6-month (None) | Depression severity, remission rate, QALYs | DSA, PSA, Scenario | vs. TAU[n]: $-8,425/QALY<br>vs. CBT[o]: $2,966/QALY | at different WTP threshold |
| Titov et al [21], 2015 | Australia | Trial-based | 12-month (None) | Depression severity, QALYs | N.R. | $4,333/QALY | |
| Philips et al [22], 2014 | U.K. | Trial-based | 12-week (None) | Depression severity, self-reported functional impairment, QALYs | N.R. | | |
| Koeser et al [23], 2013 | U.K. | Model-based (Decision-tree) | 1-year (None) | QALYs | DSA, PSA, Scenario | BtB: £-421.5 to £-190.5/QALY<br>PosMT: £-1,101.8 to £-3,445.7/QALY | £20,000 |
| *Pharmacotherapy* | | | | | | | |
| K et al [24], 2023 | India | Trial-based | 8-week (None) | Proportion of responder and remitter | Scenario | Dominant | N.R. |
| Rognoni et al [25], 2023 | Italy | Model-based (Markov) | 5-year (1-week) | Remission and response rates, QALYs | DSA, PSA, scenario | Societal: €16,314 to €22,133/QALY<br>NHS[p]: €106,705 to €111,312 | €100,000 |
| Atsou et al [26], 2021 | U.S. | Model-based (Markov) | 1-year (2-month) | Remission rate, withdrawal rate, disutility, QALYs | DSA, PSA, scenario | vs. Levomilnacipran: Dominant<br>vs. Vilazodone: $33,829/QALY | $50,000 |
| Eldar-Lissai et al [27], 2020 | U.S. | Model-based (Markov closed- | 11-year (1-week) | Remission rate, QALYs | DSA, PSA, Scenario | $106,662/QALY | $100,000 to $150,000 |

| Author (year) | Country | Modeling | Time horizon (cycle length) | Outcome measure | Sensitivity, Scenario analysis | ICER[a] | WTP[b] threshold |
|---|---|---|---|---|---|---|---|
| | | cohort state transition) | | | | | |
| Hollingworth et al [28], 2020 | U.K. | Trial-based | 12-week (None) | Depression severity, QALYs | Scenario | INMB[q]: £122 to £171/QALY | £20,000 to £30,000 |
| Wang et al [29], 2020 | China | Trial-based | 8-week (None) | Proportion of successfully treated patients | Scenario | $2,085 to $4,169/per STP[r] | $8,827 for China |
| Rubio-Valera et al [30], 2019 | Spain | Trial-based | 12-month (None) | Depression severity, QALYs | Scenario | Healthcare:<br>6m €2,549/QALY,<br>12m €6,142/QALY<br>Government:<br>6m €2,177/QALY,<br>12m €10,593/QALY | €25,000 |
| Yoon et al [31], 2019 | U.S. | Trial-based | 12-week (None) | Remission rate, QALYs | Scenario | Aug-BUP[s] vs. BUP:<br>$36,256/QALY<br>Aug-ARI[t] vs. BUP:<br>$73,295/QALY<br>Aug-ARI vs. Aug-BUP:<br>$293,620/QALY | $50,000 to $100,000 |
| Singh et al [32], 2017 | U.S. | Trial-based | 12-month (None) | Remission and response rates, QALYs | Scenario | Net health benefits were not significantly different.<br>Venlafaxine medication costs were significantly higher than bupropion and sertraline. | $30,000 |

| Author (year) | Country | Modeling | Time horizon (cycle length) | Outcome measure | Sensitivity, Scenario analysis | ICER[a] | WTP[b] threshold |
|---|---|---|---|---|---|---|---|
| Soini et al [33], 2017 | Finland | Model-based (Decision-tree /Markov) | 1-year (2-month) | Remission and relapse rates, disutility, QALYs | DSA, PSA, Scenario | Vortioxetine was a dominant ICER vs all comparators | €50,000 |
| Young et al [34], 2017 | U.K. | Model-based (Decision-tree /Markov) | 2-year (2-month) | Remission rate, disutility, QALYs | PSA, scenario | Vortioxetine was a cost-effective treatment option vs citalopram, escitalopram and sertraline. Vortioxetine was a dominant strategy vs. venlafaxine and duloxetine. | £20,000 |
| Choi et al [35], 2016 | South Korea | Model-based (Decision-tree /Markov) | 1-year (2-month) | Remission and relapse rates, disutility, QALYs | uDSA, PSA | Vortioxetine dominant | N.R. |
| Khoo et al [36], 2015 | Singapole | Model-based (Decision-analytic) | 6-month (None) | Remission and response rates, QALYs | DSA, PSA, scenario | Agomelatine was not cost effective as compared with mirtazapine (SGD 372,116/QALY) | $55,183 (SGD[u] 70,000) |
| Annemans et al [37], 2014 | Belgium | Model-based (Decision-tree) | 1-year (None) | Remission rate, QALYs | PSA, scenario | Escitalopram(€6,352/QALY) was dominated all other treatments except venlafaxine from NIHDI[v] perspective. | €30,000 |
| Maniadakis et al [38], 2013 | Greece | Model-based (Markov) | 2-year (1-month) | Remission and relapse, recurrence, discontinuation, | DSA, PSA | Agomelatine(€547/QALY) was a dominant strategy vs escitalopram, | €40,000 to €60,000 |

| Author (year) | Country | Modeling | Time horizon (cycle length) | Outcome measure | Sensitivity, Scenario analysis | ICER[a] | WTP[b] threshold |
|---|---|---|---|---|---|---|---|
| | | | | mortality rates, disutility, QALYs | | fluoxetine and sertraline, and it appeared to be cost-effective vs venlafaxine. | |
| Menacacci et al [39], 2013 | Italy | Model-based (Decision-analytic) | 1-year (None) | Remission rate, QALYs | DSA, PSA, Scenario | Escitalopram was associated with a lower cost and a larger health gain at 1year. | €25,000 |
| Menacacci et al [40], 2013 | Italy | Model-based (Decision-analytic) | 1-year (None) | Remission rate, QALYs | DSA, PSA, Scenario | Escitalopram was associated with a lower cost and a larger health gain at 1year. | €25,000 |
| Menacacci et al [41], 2013 | Italy | Model-based (Decision-analytic) | 1-year (None) | Remission rate, QALYs | DSA, PSA, Scenario | Citalopram was dominated by escitalopram. Escitalopram had an ICER of €4395 and €1080/QALY. | €25,000 |
| Saylan et al [42], 2013 | Turkey | Model-based (Markov) | Lifetime (None) | Remission rate, Major depressive episodes, QALYs | PSA | Aripiprazole provides health benefits at lower costs vs quetiapine and olanzapine augmentation | at different WTP threshold |

[a]ICER: incremental cost-effectiveness ratio.
[b]WTP: willingness to pay
[c]U.S.: united states.
[d]DFD: depression free days gain
[e]QALYs: quality-adjusted life years.
[f]PSA: probabilistic sensitivity analysis.

[g]DSA: deterministic sensitivity analysis.
[h]N.R.: not reported.
[i]EQ-5D-3L: European quality of life 5 dimensions 3 level.
[j]SF-12: 12-item short form health survey.
[k]U.K.: United Kingdom.
[l]ITT: intention to treat.
[m]CC: complete case.
[n]TAU: treatment as usual.
[o]CBT: cognitive behavioral therapy.
[p]NHS: national health service.
[q]INMB: incremental net monetary benefit.
[r]STP: successfully treated patients.
[s]Aug-BUP: augmentation bupropion
[t]Aug-ARI: augmentation aripiprazole
[u]SGD: Singapore dollar
[v]NIHDI: National Institute for Health and Disability Insurance.

1. Boggs JM, Ritzwoller DP, Beck A, Dimidjian S, Segal ZV. Cost-Effectiveness of a Web-Based Program for Residual Depressive Symptoms: Mindful Mood Balance. Psychiatr Serv. 2022;73(2):158-164. PMID:34320822
2. Langergaard A, Mathiasen K, Søndergaard J, Sørensen SS, Laursen SL, Xylander AAP, et al. Economic evaluation alongside a randomized controlled trial of blended cognitive-behavioral therapy for patients suffering from major depressive disorder. Internet Interv. 2022;28:100513. PMID:35242594
3. Piera-Jiménez J, Etzelmueller A, Kolovos S, Folkvord F, Lupiáñez-Villanueva F. Guided Internet-Based Cognitive Behavioral Therapy for Depression: Implementation Cost-Effectiveness Study. J Med Internet Res. 2021;23(5):e27410. PMID:33973857
4. Baumann M, Stargardt T, Frey S. Cost-Utility of Internet-Based Cognitive Behavioral Therapy in Unipolar Depression: A Markov Model Simulation. Appl Health Econ Health Policy. 2020;18(4):567-578. PMID:32060822
5. Gräfe V, Moritz S, Greiner W. Health economic evaluation of an internet intervention for depression (deprexis), a randomized controlled trial. Health Econ Rev. 2020;10(1):19. PMID:32548724
6. Richards D, Enrique A, Eilert N, Franklin M, Palacios J, Duffy D, et al. A pragmatic randomized waitlist-controlled effectiveness and cost-effectiveness trial of digital interventions for depression and anxiety. NPJ Digit Med. 2020;3:85. PMID:32566763
7. Thase ME, McCrone P, Barrett MS, Eells TD, Wisniewski SR, Balasubramani GK, et al. Improving Cost-effectiveness and Access to Cognitive Behavior Therapy for Depression: Providing Remote-Ready, Computer-Assisted Psychotherapy in Times of Crisis and Beyond. Psychother Psychosom. 2020;89(5):307-313. PMID:32396917
8. Kooistra LC, Wiersma JE, Ruwaard J, Neijenhuijs K, Lokkerbol J, van Oppen P, et al. Cost and Effectiveness of Blended Versus Standard Cognitive Behavioral Therapy for Outpatients With Depression in Routine Specialized Mental Health Care: Pilot Randomized Controlled Trial. J Med Internet Res. 2019;21(10):e14261. PMID:31663855
9. Yan C, Rittenbach K, Souri S, Silverstone PH. Cost-effectiveness analysis of a randomized study of depression treatment options in primary care suggests stepped-care treatment may have economic benefits. BMC Psychiatry. 2019;19(1):240. PMID:31382932
10. Holst A, Björkelund C, Metsini A, Madsen JH, Hange D, Petersson EL, et al. Cost-effectiveness analysis of internet-mediated cognitive behavioural therapy for depression in the primary care setting: results based on a controlled trial. BMJ Open. 2018;8(6):e019716. PMID:29903785
11. Kolovos S, van Dongen JM, Riper H, Buntrock C, Cuijpers P, Ebert DD, et al. Cost effectiveness of guided Internet-based interventions for depression in comparison with control conditions: An individual-participant data meta-analysis. Depress Anxiety. 2018;35(3):209-219. PMID:29329486

12. Kraepelien M, Mattsson S, Hedman-Lagerlöf E, Petersson IF, Forsell Y, Lindefors N, et al. Cost-effectiveness of internet-based cognitive-behavioural therapy and physical exercise for depression. BJPsych Open. 2018;4(4):265-273. PMID:30057780

13. Wijnen BF, Lokman S, Leone S, Evers SM, Smit F. Complaint-Directed Mini-Interventions for Depressive Symptoms: A Health Economic Evaluation of Unguided Web-Based Self-Help Interventions Based on a Randomized Controlled Trial. J Med Internet Res. 2018;20(10):e10455. PMID:30274958

14. Duarte A, Walker S, Littlewood E, Brabyn S, Hewitt C, Gilbody S, et al. Cost-effectiveness of computerized cognitive-behavioural therapy for the treatment of depression in primary care: findings from the Randomised Evaluation of the Effectiveness and Acceptability of Computerised Therapy (REEACT) trial. Psychol Med. 2017;47(10):1825-1835. PMID:28228182

15. Lee YC, Gao L, Dear BF, Titov N, Mihalopoulos C. The Cost-effectiveness of the Online MindSpot Clinic for the Treatment of Depression and Anxiety in Australia. J Ment Health Policy Econ. 2017;20(4):155-166. PMID:29300702

16. Romero-Sanchiz P, Nogueira-Arjona R, García-Ruiz A, Luciano JV, García Campayo J, Gili M, et al. Economic evaluation of a guided and unguided internet-based CBT intervention for major depression: Results from a multi-center, three-armed randomized controlled trial conducted in primary care. PLoS One. 2017;12(2):e0172741. PMID:28241025

17. Wright B, Tindall L, Littlewood E, Allgar V, Abeles P, Trépel D, et al. Computerised cognitive-behavioural therapy for depression in adolescents: feasibility results and 4-month outcomes of a UK randomised controlled trial. BMJ Open. 2017;7(1):e012834. PMID:28132000

18. Kolovos S, Kenter RM, Bosmans JE, Beekman AT, Cuijpers P, Kok RN, et al. Economic evaluation of Internet-based problem-solving guided self-help treatment in comparison with enhanced usual care for depressed outpatients waiting for face-to-face treatment: A randomized controlled trial. J Affect Disord. 2016;200:284-92. PMID:27155071

19. Geraedts AS, van Dongen JM, Kleiboer AM, Wiezer NM, van Mechelen W, Cuijpers P, et al. Economic Evaluation of a Web-Based Guided Self-Help Intervention for Employees With Depressive Symptoms: Results of a Randomized Controlled Trial. J Occup Environ Med. 2015;57(6):666-75. PMID:25741795

20. Solomon D, Proudfoot J, Clarke J, Christensen H. e-CBT (myCompass), Antidepressant Medication, and Face-to-Face Psychological Treatment for Depression in Australia: A Cost-Effectiveness Comparison. J Med Internet Res. 2015;17(11):e255. PMID:26561555

21. Titov N, Dear BF, Ali S, Zou JB, Lorian CN, Johnston L, et al. Clinical and cost-effectiveness of therapist-guided internet-delivered cognitive behavior therapy for older adults with symptoms of depression: a randomized controlled trial. Behav Ther. 2015;46(2):193-205. PMID:25645168

22. Philips R, Schneider J, Molosankwe I, Leese M, Foroushani PS, Grime P, et al. Randomized controlled trial of computerized cognitive behavioural therapy for depressive symptoms: effectiveness and costs of a workplace intervention. Psychol Med. 2014;44(4):741-52. PMID:23795621

23. Koeser L, Dobbin A, Ross S, McCrone P. Economic evaluation of audio based resilience training for depression in primary care. J Affect Disord. 2013;149(1-3):307-12. PMID:23489394

24. K G, Nagaraj M, Pavithra MS, Jyothi R, Pandith L. Randomized and parallel-group study of cost-effectiveness analysis of escitalopram and desvenlafaxine in moderate-to-severe depression. Natl J Physiol Pharm Pharmacol. 2023;13(8):1644-1649. doi:10.5455/njppp.2023.13.12618202209012022

25. Rognoni C, Falivena C, Costa F, Armeni P. Cost-Utility Analysis of Esketamine for Patients with Treatment-Resistant Depression in Italy. Pharmacoeconomics. 2023;41(2):209-225. PMID:36662417

26. Atsou K, Ereshefsky L, Brignone M, Danchenko N, Diamand F, Mucha L, et al. Cost-effectiveness of vortioxetine compared with levomilnacipran and vilazodone in patients with major depressive disorder switching from an initial antidepressant. Expert Rev Pharmacoecon Outcomes Res. 2021;21(1):29-42. PMID:33307885

27. Eldar-Lissai A, Cohen JT, Meltzer-Brody S, Gerbasi ME, Chertavian E, Hodgkins P, et al. Cost-Effectiveness of Brexanolone Versus Selective Serotonin Reuptake Inhibitors for the Treatment of Postpartum Depression in the United States. J Manag Care Spec Pharm. 2020;26(5):627-638. PMID:32191592

28. Hollingworth W, Fawsitt CG, Dixon P, Duffy L, Araya R, Peters TJ, et al. Cost-Effectiveness of Sertraline in Primary Care According to Initial Severity and Duration of Depressive Symptoms: Findings from the PANDA RCT. Pharmacoecon Open. 2020;4(3):427-438. PMID:31777008

29. Wang G, Zhao K, Reynaud-Mougin C, Loft H, Ren H, Eriksen HF, et al. Successfully treated patients with vortioxetine versus venlafaxine: a simplified cost-effectiveness analysis based on a head-to-head study in Asian patients with major depressive disorder. Curr Med Res Opin. 2020;36(5):875-882. PMID:31990207

30. Rubio-Valera M, Peñarrubia-María MT, Iglesias-González M, Knapp M, McCrone P, Roig M, et al. Cost-effectiveness of antidepressants versus active monitoring for mild-to-moderate major depressive disorder: a multisite non-randomized-controlled trial in primary care (INFAP study). Eur J Health Econ. 2019;20(5):703-713. PMID:30725226

31. Yoon J, Zisook S, Park A, Johnson GR, Scrymgeour A, Mohamed S. Comparing Cost-Effectiveness of Aripiprazole Augmentation With Other "Next-Step" Depression Treatment Strategies: A Randomized Clinical Trial. J Clin Psychiatry. 2018;80(1):18m12294. PMID:30695291

32. Singh A, Brooks MM, Voorhees RE, Potter MA, Roberts MS, Luther JF, et al. Cost-Effective Drug Switch Options After Unsuccessful Treatment With an SSRI for Depression. Psychiatr Serv. 2017;68(1):81-87. PMID:27524365

33. Soini E, Hallinen T, Brignone M, Campbell R, Diamand F, Cure S, et al. Cost-utility analysis of vortioxetine versus agomelatine, bupropion SR, sertraline and venlafaxine XR after treatment switch in major depressive disorder in Finland. Expert Rev Pharmacoecon Outcomes Res. 2017;17(3):293-302. PMID:27680105

34. Young AH, Evitt L, Brignone M, Diamand F, Atsou K, Campbell R, et al. Cost-utility evaluation of vortioxetine in patients with Major Depressive Disorder experiencing inadequate response to alternative antidepressants in the United Kingdom. J Affect Disord. 2017;218:291-298. PMID:28478358

35. Choi SE, Brignone M, Cho SJ, Jeon HJ, Jung R, Campbell R, et al. Cost-effectiveness of vortioxetine versus venlafaxine (extended release) in the treatment of major depressive disorder in South Korea. Expert Rev Pharmacoecon Outcomes Res. 2016;16(5):629-638. PMID:26641142

36. Khoo AL, Zhou HJ, Teng M, Lin L, Zhao YJ, Soh LB, et al. Network Meta-Analysis and Cost-Effectiveness Analysis of New Generation Antidepressants. CNS Drugs. 2015;29(8):695-712. PMID:26293743

37. Annemans L, Brignone M, Druais S, De Pauw A, Gauthier A, Demyttenaere K. Cost-effectiveness analysis of pharmaceutical treatment options in the first-line management of major depressive disorder in Belgium. Pharmacoeconomics. 2014;32(5):479-93. PMID:24554474

38. Maniadakis N, Kourlaba G, Mougiakos T, Chatzimanolis I, Jonsson L. Economic evaluation of agomelatine relative to other antidepressants for treatment of major depressive disorders in Greece. BMC Health Serv Res. 2013;13:173. PMID:23663281

39. Mencacci C, Aguglia E, Biggio G, Cappellari L, Di Sciascio G, Fagiolini A, et al. C-QUALITY: cost and quality-of-life pharmacoeconomic analysis of antidepressants in major depressive disorder in Italy. Adv Ther. 2013;30(7):697-712. PMID:23929174

40. Mencacci C, Aguglia E, Biggio G, Cappellari L, Di Sciascio G, Fagiolini A, et al. C-QUALITY: cost and quality-of-life pharmacoeconomic analysis of antidepressants used in major depressive disorder in the regional Italian settings of Veneto and Sardinia. Clinicoecon Outcomes Res. 2013;5:611-21. PMID:24348057

41. Mencacci C, Di Sciascio G, Katz P, Ripellino C. Cost-effectiveness evaluation of escitalopram in major depressive disorder in Italy. Clinicoecon Outcomes Res. 2013;5:87-99. PMID:23413176

42. Saylan M, Treur MJ, Postema R, Dilbaz N, Savas H, Heeg BM, et al. Cost-Effectiveness Analysis of Aripiprazole Augmentation Treatment of Patients with Major Depressive Disorder Compared to Olanzapine and Quetiapine

Augmentation in Turkey: A Microsimulation Approach. Value Health Reg Issues. 2013;2(2):171-180. PMID:29702862
